# Towards Understanding the Genetic Nature of Vasovagal Syncope

**DOI:** 10.3390/ijms221910316

**Published:** 2021-09-24

**Authors:** Natalia Matveeva, Boris Titov, Elizabeth Bazyleva, Alexander Pevzner, Olga Favorova

**Affiliations:** 1 National Medical Research Center for Cardiology, 121552 Moscow, Russia; natalijamat70@gmail.com (N.M.); titovborisvas@gmail.com (B.T.); dr.fedorova.ea@gmail.com (E.B.); avpevzner@mail.ru (A.P.); 2 Department of Molecular Biology and Medical Biotechnology, Pirogov Russian National Research Medical University, 117997 Moscow, Russia

**Keywords:** syncope, vasovagal syncope, genetics, complex disorders, susceptibility, twin studies, family studies, candidate gene association studies, genome-wide studies

## Abstract

Syncope, defined as a transient loss of consciousness caused by transient global cerebral hypoperfusion, affects 30–40% of humans during their lifetime. Vasovagal syncope (VVS) is the most common cause of syncope, the etiology of which is still unclear. This review summarizes data on the genetics of VVS, describing the inheritance pattern of the disorder, candidate gene association studies and genome-wide studies. According to this evidence, VVS is a complex disorder, which can be caused by the interplay between genetic factors, whose contribution varies from monogenic Mendelian inheritance to polygenic inherited predisposition, and external factors affecting the monogenic (resulting in incomplete penetrance) and polygenic syncope types.

## 1. Introduction

Syncope, or fainting, is characterized by global cerebral hypoperfusion, transient loss of consciousness with disturbed postural tone, disturbance of the cardiovascular and respiratory systems, and spontaneous recovery back to the normal state [1].

A reduction in systemic blood pressure (BP) causing a decrease in cerebral blood flow plays a major role in the pathogenesis of syncope [2]. In turn, systemic BP depends on cardiac output and systemic peripheral vascular resistance [2,3]. The cardiac output and peripheral vascular resistance are affected by many factors such, as autonomic dysfunction, various cardiovascular (CV) diseases, decreased venous return, etc. The classification of syncope is based on these and other factors. According to the European Society of Cardiology Guidelines for the diagnosis and management of syncope [1], the following subtypes of syncope are distinguished: reflex syncope, cardiac syncope, and syncope due to orthostatic hypotension.

Approximately 40% of humans during their lifetime have transient loss of consciousness [4]; two-thirds of them are reflex syncope (also known as neurally mediated syncope) [5]. Vasovagal syncope (VVS) is the most common type of fainting in this group. A person develops VVS due to abnormal autonomic control of blood circulation, when sympathetic tone is decreased and the parasympathetic nervous system temporarily becomes overactive, thus resulting in arterial hypotension and cerebral hypoperfusion. VVS is often accompanied by bradycardia, and in some cases, by prolonged asystole [6]. VVS can be induced by various triggers such as orthostatic stress, exposure to emotional stress, medical manipulations, etc., and has such autonomic symptoms as hot flushes and nausea followed by dizziness and transient loss of consciousness. Although the outcome of VVS is favorable, this condition significantly worsens quality of life and can cause physical and mental injury. VVS more likely occurs at a young age, although cases when it first occurs in middle-aged and elderly patients have also been reported [7]. There are sex-specific differences in the prevalence and clinical manifestations of VVS: females are 50% more likely to have premonitory signs and symptoms of VVS than males [8].

A surrogate marker, the head-up tilt test (or prolonged passive head up tilt testing) has been used to diagnose VVS since the late 1980s. In this test, the patient is moved from the horizontal to vertical position using a special tilting table in order to simulate the neurally mediated reflex and induce syncope. The probability that VVS is induced during the head-up tilt test is 40–60%. The positive head-up tilt test response is associated with frequent VVS episodes and may be indicative of significant severity of orthostatic disorders [9]. Meanwhile, a negative head-up tilt test does not always mean that an individual cannot be diagnosed with neurally mediated syncope. This diagnosis should be suspected in individuals with typical clinical manifestations once all other reasons for transient loss of consciousness have been eliminated. The causes and the mechanism of VVS development have not been fully elucidated yet. This review describes and analyzes the publications focusing on the contribution of genetic components to the development of VVS. Having identified the genetic risk factors, one obtains efficient tools for performing a personalized prognosis of susceptibility to VVS. Furthermore, the molecular foundations of VVS pathogenesis can be revealed to develop new strategies for its prevention and management.

## 2. The Inheritance Pattern of VVS

The first step in studying hereditary diseases is often to perform a familial aggregation analysis. If familial aggregation is revealed, the patient’s relatives have a higher risk of developing the disease compared to the average risk within a population, which is inversely proportional to the genetic distance from the proband. The earliest studies focused on the inheritance of VVS have identified familial aggregation of syncope. Kleinknecht and Lenz [10] reported that 66% of students that faint as a result of seeing blood or injuries on themselves or on others had at least one parent with VVS, while 41% of non-fainters had parents with VVS (*p* < 0.01). Among individuals with VVS unrelated to medical triggers, 94% of subjects had a family history of syncope [11].

In an early study, the family history of 30 children with VVS was examined [12]. For 24 of these children, their family histories were compared to those of their best friends (the control group). In these two groups, 90% of children with VVS and only 33% of children in the control group (*p* < 0.01) had at least one first degree relative also suffering from VVS. Furthermore, 37% of children with VVS had both a sibling and a parent with syncope vs. 4% of controls (*p* < 0.05). It was inferred that VVS is a complex disorder with both inherited genetic and external factors involved in its development.

In a study [13] involving 441 patients with VVS confirmed by the head-up tilt test, 19% of patients were found to have a familial history of this condition. It turned out that 37.2% of relatives of these patients also had VVS episodes. The researchers drew the conclusion that VVS undoubtedly has a genetic component and suggested that VVS either is an autosomal recessive disorder or its inheritance pattern is complex (does not strictly obey the Mendelian inheritance pattern).

Studying family history records identified families in which several generations suffered from VVS [14,15]. Thus, the study [15] presented a case report of a family consisting of three generations where all nine family members suffered from syncope. In this family, the inheritance pattern of VVS coincided with that expected in cases of autosomal dominant inheritance with incomplete penetrance.

Therefore, family aggregation studies of VVS inevitably demonstrated that probands with VVS were more likely to have a positive family history compared to the control group. A number of researchers have put forward a skeptical hypothesis that family aggregation might be observed randomly due to the high syncope rate among the population [16,17]; other scholars, however, do not support this point of view [18].

Twin studies allow one to examine the contribution of genetics to the development of a disease/phenotype by comparing the concordance in monozygotic and dizygotic twins. The first twin study focusing on syncope was conducted in 659 twin pairs from the Australian Twin Registry with respect to syncope related to blood/injury/injection fear [19]. A significant family aggregation of this type of syncope was observed; however, statistical methods could not discern contributions of the genetic component and overall external factors as the reason for fainting. In another study conducted on 51 monozygotic twin pairs from the Australian Twin Registry where at least one twin experienced syncope, a higher concordance among monozygotic twins compared to dizygotic ones was observed [20]. Significant effects were revealed both for syncope unrelated to external factors (*p* = 0.018) and for syncope related to typical vasovagal triggers (sight of blood, injuries, medical manipulations, standing too long in one place, or pain) (*p* < 0.001). The results of this study are also consistent with the assumption that VVS is a complex disorder, with both genetic and environmental factors contributing to its development. According to [20], the number of close relatives suffering from VVS complies with an autosomal dominant inheritance pattern in 7 out of 19 pairs of concordant monozygotic twins.

A total of 2,694,442 subjects from several Swedish nationwide registries were enrolled in a recent large-scale study [21], including 1,570,128 siblings, out of whom 24,020 subjects were twins; 264,244 subjects were half-siblings; and 1,044,546 subjects were cousins. The risk of syncope among relatives with VVS was maximal in twins and decreased systematically depending on the genetic distance from the proband.

The reported data are indicative of the role played by the genetic component in the development of VVS. However, as mentioned in the review by Sheldon and Sandhu [17], a significant number of cited studies have drawbacks such as the lack of clearly defined diagnostic criteria for the formation of initial study groups and/or control groups and the use of approaches based on self-assessment of patients whose recollections can be biased (especially with respect to the family history of syncope).

The effect of sex and age on the risk of VVS can be another reason for erroneous results. Indeed, a study enrolling 62 medical students and their families reported that females were more likely to experience syncope than males [22]. By the age of 30 years, the risk of syncope for females and males was 34% and 10%, respectively, if both their parents had no VVS episodes. This parameter increased to 48% and 28% for females and males, respectively, if one of the subjects’ parents had VVS, and was as high as 78% (females) or 55% (males) if both parents had VVS. A positive maternal history of VVS increased the risk of syncope threefold in both male and female descendants, while a positive paternal history of VVS increased the risk only for male descendants. Similar data were obtained using the proportional hazards model [14]. It was shown that both male and female descendants with maternal history of VVS were more likely to experience syncope than those whose mother was a non-fainter, while the paternal history of syncopal episodes significantly increased the risk of VVS in sons but not in daughters. Interesting observations were made regarding the family history of three concordant monozygotic twin pairs with recurrent VVS [14,23]. The mother of a pair of twin girls also suffered from syncope [14], while both parents of the two pairs of twin boys were non-fainters [14,23]. The risk of VVS increases with age: the first syncopal episode occurs in most patients by age 30 years, followed by syncope recurrence over the next decades [7].

Hence, the individual susceptibility to VVS largely depends on one’s sex and age, as well as on the sex of the parent who suffers from VVS. These data are consistent with the theory of a potential contribution of epigenetic factors to the development of VVS. Epigenetic regulation is not related to nucleotide sequence changes in the genome, but affects the transcriptional level of the genes important for phenotype development via covalent modification of DNA or histone proteins. Since sex hormones can modulate gene expression, the differences in prevalence of VVS in males and females can be attributed to the sex-hormone-dependent epigenetic mechanisms.

Overall, the observed complex inheritance pattern demonstrates that VVS has complex (multifactorial) origins: its development can be regulated by interplay between the genetic and epigenetic, as well as environmental factors. Importantly, environmental factors can modulate epigenetic processes [24], and these two mechanisms are not alternative in real practice. Meanwhile, taking into account the clinical heterogeneity of VVS and data variation in selected publications on family history, pedigrees, and results of twin studies, it is quite likely that the contribution of environmental factors and the inherited genetic component varies within a broad range. In turn, the genetic component can vary from monogenic Mendelian inheritance (with autosomal recessive or autosomal dominant patterns) to polygenic inherited predisposition.

What is the ratio between the different inheritance patterns of VVS? The findings reported in [25] provide a rough idea of this. Among the 44 multiplex families with VVS examined in [25], an autosomal dominant inheritance pattern was revealed in 6 families. The largest of these families included 30 subjects suffering from VVS in 3 generations; in the remaining 5 families, the number of subjects with VVS ranged from 4 to 14.

## 3. Candidate Gene Association Studies

The conventional approach to searching for genes involved in disease/phenotype development still remains relevant and is based on analyzing the association of individual candidate genes with the phenotype. An assumption that a gene is possibly associated with the phenotype is made based on the function of the gene product (the “phenotype-to-gene” approach). According to views on pathogenesis of VVS, one can expect that the genes whose products regulate the functioning of the autonomic nervous system and the cardiovascular system are mainly involved in syncope development. It is worth mentioning that genes are ascribed to either of these categories rather tentatively, since the autonomic nervous system regulates the function of all internal organs, including the heart and blood vessels, while variations in functioning of the cardiovascular system trigger a response from the nervous system. In most cases, researchers analyze polymorphic variants of genes, represented by single nucleotide polymorphisms (SNPs).

Data obtained from case-control studies on the association between carriership of polymorphic variants of a certain candidate gene and susceptibility to VVS are summarized in Table 1. The genotype frequencies in patients with VVS and non-fainting controls were compared in almost 50% of the studies. In the remaining studies, a surrogate marker was used instead of controls: patients susceptible and not susceptible to induction of VVS by the head-up tilt test were employed. Undoubtedly, this comparison can be reasonably performed, but it is not equivalent to direct comparison of the presence or absence of VVS. Indeed, as one can see in Table 1, the identified associations between a gene and the head-up tilt test response often mismatch the data obtained by comparing patients and controls. 

Before we discuss the results obtained by assessing the involvement of individual genes in the development of VVS, we would like to point out the methodological flaws that some of the cited studies have. In a conventional association study, the groups being compared need to be characterized according to the ESC Guidelines for the diagnosis and management of syncope [4]. However, in actual practice, the study groups (and especially the control one) were often formed using questionnaire data only. The study group could include individuals with suspected VVS rather than those with a definitive diagnosis [26], or include patients with a history of both typical and atypical VVS [27]. In individual studies, patients and controls in the groups being compared were members of the same families, which does not meet the sample independence criterion [28]. Finally, the sample size was often insufficient: the number of probands with VVS ranged from 50 [29] to 347 individuals [30], while the number of controls ranged from 32 [31] to 150 individuals [32]. The publication that stands apart is study [33], where population-wide data from an earlier study [34] was used as the control group. Although a study containing large control groups is certainly appealing, this method generates doubts when taking into account the high population-wide frequency of VVS.

**Table 1:** Data on the association of polymorphic loci of candidate genes with vasovagal syncope (VVS).

Gene Symbol (Protein)	Rs Number (Amino Acid Substitution)	Ref.	Probands with VVS, N			Controls (without VVS, Ctrl), N	*p* Value (Compared Groups)	Note
			Total	Tilt +	Tilt −			
Genes of the adrenergic receptors								
*ADRA1A* (Alpha 1— adrenergic receptor)	rs1048101 (Arg347Cys)	[35]	89	89	0	40	**<0.001** **(VVS vs. Ctrl)**	All controls were with a negative tilt test
		[28]	82	*	*	78	NS (VVS vs. Ctrl)	
		[36]	129	73	56	0	NS (Tilt+ vs. Tilt−)	
		[37]	134	88	46	0	NS (Tilt+ vs. Tilt−)	
	rs1383914 rs574584 rs573542	[37]	134	88	46	0	NS (Tilt+ vs. Tilt−)	
*ADRB1* (Beta 1 adrenergic receptor)	rs1801253 (Arg389Gly)	[29]	50	33	17	0	**0.012** **(Tilt+ vs. Tilt−)**	
		[38]	70	48	22	0	**0.001** **(Tilt+ vs. Tilt−)**	
		[36]	129	73	56	0	NS (Tilt+ vs. Tilt−)	Also no association with the number of new syncope
		[39]	205	95	110	143	NS (VVS **vs.** C)	
		[28]	82	*	*	78	NS (VVS **vs.** C)	
		[37]	134	88	46	0	NS (Tilt+ vs. Tilt−)	
		[40]	123	123	0	0	**0.012** **(Arg389Arg vs. Arg389Gly)**	Association with the number of syncope
	rs1801252 (Ser49Gly)	[36]	129	73	56	0	NS (Tilt+ vs. Tilt−)	Also no association with the number of new syncope episodes
		[28]	82	*	*	78	NS (VVS **vs.** Ctrl)	
		[37]	134	88	46	0	**0.02** **(Tilt+ vs. Tilt−)**	
*ADRB2* (Beta 2 adrenergic receptor)	rs1042713 (Gly16Arg)	[36]	129	73	56	0	NS (Tilt+ vs. Tilt−)	
		[37]	134	88	46	0	**0.04** **(Tilt+ vs. Tilt−)**	
	rs1042714 (Gln27Glu)	[36]	129	73	56	0	NS (Tilt+ vs. Tilt−)	
		[37]	134	88	46	0	NS (Tilt+ vs. Tilt−)	
*ADRB3* (Beta 3 adrenergic receptor)	rs4994	[37]	134	88	46	0	NS (Tilt+ vs. Tilt−)	
Genes of the serotonin signaling								
*SLC6A4* (Serotonin transporter)	rs25531	[41]	191	117	74	0	NS (Tilt+ vs. Tilt−)	
	rs4795541	[36]	129	73	56	0	NS (Tilt+ vs. Tilt−)	
	no rs; 43-bp insertion/deletion in the promoter region	[28]	82	*	*	78	NS (VVS **vs.** Ctrl)	There was a trend towards gender-specific differences in the effect of alleles (*p* > 0.05)
*HTR1A* (Serotonin 5-HT1A receptor)	rs6295	[28]	82	*	*	78	**0.005** **(VVS vs. Ctrl,** **only males)**	
*COMT* (Catechol O-methyl- transferase)	rs4680	[28]	82	*	*	78	**0.017** **(VVS vs. Ctrl,** **with** **gender-specific allele effect)**	
Genes of the adenosine receptors								
*ADORA2A* (Adenosine A2A receptor)	rs5751876 (Tyr361Tyr)	[42]	105	52	53	121	**<0.0001** **(Tilt+ vs. Tilt−)** NS (VVS **vs.** Ctrl)	There was also an association with the frequency of syncope episodes
		[30]	347	207	140	83	NS (VVS **vs.** Ctrl) NS (Tilt+ vs. Tilt−)	An association was observed with heart rate in the early phase of tilt and during syncope
		[28]	82	*	*	78	NS (VVS **vs.** Ctrl)	
G protein signaling genes								
*GNAS1* (G protein alpha subunit)	rs7121 C393T (silent mutation Ile131)	[43]	137	96	41	**0**	**<0.001** **(Tilt+ vs. Tilt−)**	
		[44]	307	207	100	74	NS (VVS **vs.** Ctrl) NS (mild vs. malignant syncope)	
		[28]	82	*	*	78	NS (VVS **vs.** Ctrl)	
*GNB1* (G protein beta 1 subunit)	rs17363334 rs77354509 rs79516120	[45]	74	74	0	208	NS (VVS vs. Ctrl)	
*GNB3* (G protein beta 3 subunit)	rs5443 C825T leads to alternative splicing with a loss of 41 amino acids	[27]	68	68	0	0	**<0.001** **(typical vs. non-typical vasovagal history)**	56 patients with typical VVS history and 12—with non-typical VVS history
		[31]	213	*	*	32	NS (VVS **vs.** Ctrl)	All controls were with a negative tilt test
		[44]	307	207	100	74	NS (VVS **vs.** Ctrl) NS (mild vs. malignant syncope)	
		[46]	217	152	65	0	NS (Tilt+ vs. Tilt−)	
		[36]	129	73	56	0	NS (Tilt+ vs. Tilt−)	
		[26]	157	91	66	109	NS (VVS **vs.** Ctrl) NS (Tilt+ vs. Tilt−)	Patients with suspected VVS
*GNG2* (G protein gamma 2 subunit)	no rs; c.87 + 34G > A	[45]	74	74	0	208	NS (VVS vs. Ctrl)	
*RGS2* (G protein signaling regulator)	rs4606 C1114G	[46]	217	152	65	0	NS (Tilt+ vs. Tilt−)	
		[44]	307	207	100	74	NS (VVS **vs.** Ctrl) NS (syncope severity)	
		[47]	214	145	69	40	**0.04** **(different number of syncope** **episodes)**	
		[32]	300	150	150	150	NS (VVS **vs.** Ctrl) NS (Tilt+ vs. Tilt−)	
Genes of the potassium channels								
*KCNJ5* (Inwardly rectifying potassium channel, subfamily J, member 5)	rs45516097	[45]	74	74	0	208	**0.001** **(VVS vs. Ctrl)**	Minor allele T is less common in patients with VVS
	rs6590357 rs7118824 rs7118833 rs7102584 rs4937391	[45]	74	74	0	208	NS (VVS vs. Ctrl)	
*KCNJ3* (Inwardly rectifying potassium channel, subfamily J, member 3)	rs16838016 rs3111033 rs17642086 rs80085601	[45]	74	74	0	208	NS (VVS vs. Ctrl)	
*KCNH2* (Voltage-gated potassium channel subfamily H member 2)	rs1805123	[28]	82	*	*	78	NS (VVS vs. Ctrl)	
*KCNE1* (Voltage-gated potassium channel subfamily E member 1)	rs1805127	[28]	82	*	*	78	NS (VVS vs. Ctrl)	
Genes encoding vasoactive proteins								
*ACE* (Angiotensin- converting enzyme)	rs4646994 insertion/ deletion of the Alu repeat	[33]	165	165	0	>6000	NS (VVS vs. Ctrl)	Control data according to [34]
		[41]	191	117	74	0	NS (Tilt+ vs. Tilt−)	
*AGT* (Angiotensinogen)	rs699	[41]	191	117	74	0	NS (Tilt+ vs. Tilt−)	
*AGTR1* (Angiotensin II receptor Type 1)	rs5186	[41]	191	117	74	0	NS (Tilt+ vs. Tilt−)	
*eNOS* (Endothelial NO synthase 3)	rs2070744 rs1799983	[28]	82	*	*	78	NS (VVS vs. Ctrl)	
*EDNRA* (Endothelin type A receptor)	rs5333	[48]	107	58	49	208	NS (VVS vs. Ctrl) NS (Tilt+ vs. Tilt−)	
*EDN1* (Endothelin 1)	rs1800997 insertion/ deletion 3A/4A	[48]	107	58	49	208	NS (VVS vs. Ctrl) **0.048** **(Tilt+ vs. Tilt−)**	Allele 4A is associated with a positive tilt test
Other genes								
*DBH* (Dopamine beta hydroxylase)	rs1611115	[36]	129	73	56	0	NS (Tilt+ vs. Tilt−)	
*CHRM2* (Muscarinic M2 receptor)	rs138806839 c.1114C > G	[45]	74	74	0	208	NS (VVS vs. Ctrl)	

Tilt+: VVS patients with a positive tilt test. Tilt−: VVS patients with a negative tilt test. Associations are considered significant at *p* < 0.05 and highlighted in bold. NS—non-significant. * Data not provided.

Since such neurotransmitters as norepinephrine, epinephrine, and serotonin are believed to play a major role in the development of syncope, genetic factors of susceptibility to VVS are primarily searched for among genes encoding receptors, carrier proteins, and enzymes partaking in the synthesis of these mediators.

The genes encoding adrenergic receptors are the most interesting candidate genes for VVS; the contribution of polymorphic variants of these genes was studied both to the development of VVS and head-up tilt test response. In the *ADRA1A* gene encoding the alpha-1A adrenergic receptor, the SNP rs1048101 (1039T > C) is responsible for a Cys347Arg substitution at the C-terminal end of alpha-1 adrenergic receptor; this substitution can affect receptor–protein interactions and, therefore, signal transduction from the receptor to the cell. Hernández-Pacheco et al. compared groups consisting of 89 tilt-positive patients and 40 healthy tilt-negative subjects without a history of VVS, heart or lung disease, and revealed a positive association between VVS and carriership of the C allele and the CC genotype (i.e., the presence of Arg347 in the protein) (*p* < 0.001) [35]. The authors suggest that Arg347 accelerates receptor internalization and therefore reduces the intracellular concentration of calcium ions, causing vasodilation and reducing venous return, thus increasing the risk of BP reduction and the development of VVS. In another study, a comparison of 82 patients with VVS with 79 healthy controls without structural or ECG cardiac abnormalities originating from one of nine families did not identify this association (this sample was used to analyze another 11 polymorphisms) [28]. The researchers put forward a hypothesis that rs1048101 (Arg347Cys) is associated with a positive head-up tilt test response rather than with VVS, although their samples were not characterized using the head-up tilt test. However, this assumption is not consistent with data obtained in studies where the polymorphic variants of the *ADRA1A* gene were compared in tilt-positive and tilt-negative patients with VVS. Thus, Sorrentino et al. [36] revealed no association between *ADRA1A* rs1048101 and head-up tilt response in 129 patients suffering from VVS without a history of cardiovascular disease or carotid sinus syndrome who were not taking medications affecting the cardiovascular system. A recent study observed differences in allele/genotype frequencies for neither rs1048101 nor other SNPs of the *ADRA1A* gene (rs1383914, rs574584, and rs573542) when comparing 88 tilt-positive and 46 tilt-negative patients with VVS [37].

Most of the studies listed in Table 1 focus on the contribution of variants of the beta 1 adrenergic receptor gene *ADRB1* to the development of VVS. This gene mediates positive chronotropic and inotropic effects in the cardiac muscle tissue and acts as a target for beta blockers. Researchers focused on two SNPs in the coding region of the *ADRB1* gene, rs1801253 (Arg389Gly) and rs1801252 (Ser49Gly), which affect the receptor function and its response to adrenoblockers. The Arg389Gly polymorphism resides in the C-terminal region of the beta 1 adrenergic receptor, affects its binding to G protein and, thereby, activation of adenylate cyclase. The Arg389 variant was shown to stimulate adenylate cyclase more efficiently and enhances signal transduction from adrenergic receptors compared to Gly389 [49]. The Ser49Gly polymorphism resides in the extracellular domain of the protein; the Gly49 variant enhances receptor desensitization after exposure to agonist and reduces receptor activity [50]. Comparison of patients suffering from VVS and the controls without previous history of fainting revealed no association between the SNP rs1801253 or SNP rs1801252 and VVS [28,39]. However, associations have been detected when comparing tilt-positive and tilt-negative patients. Thus, an association between the rs1801253(G) allele and positive head-up tilt test response (*p* = 0.012) [29] and, showing a good agreement with these data, an association between the CC genotype and negative head-up tilt test response (*p* < 0.001) were revealed [38]. However, a more recent study employing a larger sample replicated the data on association only between the SNP rs1801253 (but not SNP rs1801252) and the positive head-up tilt test response (*p* = 0.02) [37]. Sorrentino et al. [36] found no association between both of these polymorphic regions and head-up tilt test response. Special mention should be made of the study where the association of genotypic and phenotypic traits in 123 tilt-positive patients who had at least three syncopal episodes over one year was assessed [40]. Patients with the CC genotype (Arg389Arg) had a much higher number of syncopal episodes (*p* = 0.012); these patients also showed a better response to beta blocker therapy compared to those with the CG genotype (Arg389Gly) (*p* < 0.001).

For variants of the beta 2 adrenergic receptor gene, *ADRB2*, a comparison was performed only between tilt-positive and tilt-negative patients with VVS. No association of *ADRB2* rs1042713 or *ADRB2* rs1042714 with head-up tilt test response was found [36]. Márquez et al. [37] also found no association between rs1042714 and head-up tilt test response but observed this association for rs1042713 (*p* = 0.04). They also found an association between rs4994 in the *ADRB3* gene encoding beta 3 adrenergic receptor and the head-up tilt test response (*p* = 0.03).

Therefore, data on contribution of polymorphic variants of genes encoding alpha and beta adrenergic receptors to genetic susceptibility to VVS are rather controversial. When patients with VVS were compared to healthy controls, only an association between *ADRA1A* rs1048101 and VVS was observed [35], whereas no associations were detected when making other similar comparisons (including one more study for rs1048101 [28]). When analyzing the association between polymorphic variants of the adrenergic receptor genes and head-up tilt test response, an association was observed for 4 out of 13 comparisons.

The previously mentioned study [36] focused not only on SNPs of the adrenergic receptor genes, but also on the rs1611115 variant of the *DBH* gene encoding dopamine beta-hydroxylase, which catalyzes conversion of dopamine to norepinephrine. Comparison of tilt-positive and tilt-negative patients with VVS found no association between this SNP and head-up tilt test response (the data are provided in the “Other genes” section of Table 1).

There currently is no agreement regarding the role of serotonin in VVS development. An assumption was made that since serotonin is related to BP regulation and is found in the brain regions involved in the development of VVS, this neurotransmitter may contribute due to the antisympathetic effects mediated by the central nervous system [51]. Table 1 summarizes data on the association of head-up tilt test response and the *SLC6A4*, *HTR1A*, and *COMT* genes, whose products participate in the serotoninergic system. No differences in the carriership of polymorphic variants of rs25531 [41] and rs4795541 [36] in the serotonin transporter gene *SLC6A4* between tilt-positive and tilt-negative patients with VVS were found. Negative results were also received for the insertion/deletion polymorphism L/S (43 bp Ins/Del) within the promoter of this gene for patients with VVS compared to healthy controls [28].

A study [28] conducted using familial data reported interesting findings on sex-related differences in the contribution of the genes of the serotoninergic system to the development of VVS. Association of the G allele of the SNP rs6295 (1019G > C) of the serotonin 1a receptor gene (*HTR1A*) with VVS was revealed only in males (*p* = 0.005). This polymorphic variant resides in the promoter region of the gene directly within the binding site of the transcription factor NUDR, which can affect the receptor expression level [52]. As reported in the study [28], the A allele of the SNP rs4680 (472G > A, Val158Met) of the *COMT* gene encoding catechol-O-methyl transferase related to a reduction in the enzyme level was associated with a lower risk of VVS in males and higher risk in females (*p* = 0.017). The *COMT* gene is known to have different effects on cerebral function and dysfunction in males and females, and is involved in sex-specific dimorphism of susceptibility to mental disorders [53]. It should be mentioned here that catechol-O-methyl transferase catalyzes the degradation of dopamine, as well as epinephrine, norepinephrine, and catechol estrogens; it is not directly related to the serotoninergic system. However, the reduced level of this enzyme increases the concentration of dopamine, which competes with serotonin for transport proteins capable of carrying monoamines, and disrupts serotoninergic regulation. Sheldon et al. [28] observed a similar trend of sex specificity for the L allele of the insertion/deletion polymorphism L/S in the *SLC6A4* gene; however, the differences did not reach statistical significance (*p* = 0.059). A conclusion has been drawn that males carrying any of the aforementioned three allelic variants of the genes involved in serotoninergic regulation are protected against VVS compared to females and to other males not carrying these allelic variants. Undoubtedly, these results need to be reproduced using independent samples.

A large group of candidate genes refers to the genes whose products are involved in functioning of the cardiovascular system (regulation of cardiac rhythm and vascular tone, as well as BP maintenance). Special focus is placed on the genes whose products participate in purinergic signal transduction (adenosine receptors), nitric oxide metabolism, functioning of potassium channels, and of the renin–angiotensin–aldosterone system.

The association of the SNP rs5751876 in the *ADORA2A* gene encoding adenosine A2A receptor with susceptibility to VVS, as well as to induction of VSS during the head-up tilt test, was investigated in three publications. The study [42], which included 105 patients with a history of at least two syncopal or presyncopal episodes over the preceding year and 121 healthy controls, identified an association of the CC genotype of rs5751876 with a positive head-up tilt test response in VVS patients (*p* < 0.0001), as well as with a high frequency of syncopal episodes (*p* = 0.004), however, no association with susceptibility to VVS has been found. The SNP rs5751876 is responsible for the synonymous substitution Tyr361Tyr. The researchers suggested that this polymorphism may affect gene expression level and protein folding. In a more recent study, analyzing a much greater number of patients (n = 347) with a history of at least one syncopal episode of unknown etiology and 85 controls without a history of syncopal episodes, no association of this SNP with head-up tilt test response or VVS was detected [30]. Sheldon et al. [28] also observed no association of the SNP rs5751876 with susceptibility to VVS.

Many receptors involved in signal transduction in patients suffering from VVS belong to the group of G protein-coupled receptors. The potential role of genes encoding G proteins in the formation of genetic predisposition to VVS has mainly been investigated by Lelonek et al. In the series of studies, they analyzed the SNP rs7121 of the *GNAS1* gene encoding protein G subunit alpha, the SNP rs5443 of the *GNB3* gene encoding protein G subunit beta 3, and the SNP rs4606 of the *RGS2* gene encoding the G protein signaling regulator 2; these SNPs have also been analyzed by other researchers. A comparison of VVS patients with healthy controls revealed that none of these genes are associated with VVS [31,46,47]. Other researchers obtained similar results for the *GNAS1* [28], *GNB3* [26], and *RGS2* genes [32]; the sample size of the group analyzed in [32] was rather large (300 children with VVS and 150 healthy children). Furthermore, no association of the SNPs rs17363334, rs77354509, or rs79516120 of the *GNB1* gene encoding G protein subunit beta 1 and c.87 + 34G > A of the *GNG2* gene encoding G protein subunit gamma 2 with VVS was detected [45]. No association between polymorphisms of the *GNAS1* and *GNB3* genes and syncope severity was revealed in patients with a history of more than three syncopal episodes over the preceding two years [44]. Meanwhile, polymorphisms in the *GNB3* gene were found to be associated with a history of VVS (typical VVS vs. atypical VVS) (*p* < 0.001) [27]; polymorphisms in the *RGS2* gene were found to be associated with the number of syncopal episodes (*p* = 0.04) [47]. Out of five studies focusing on the association between the G protein genes (*GNAS1*, *GNB3* or *RGS2*) and head-up tilt test response, such an association was found only in study [43] for the SNP rs7121 of the *GNAS1* gene (*p* < 0.001). 

Hence, the best reproducibility of findings has been achieved by analyzing the association between G protein genes and VVS. These data allow one to infer that the analyzed polymorphic variants of the G protein genes have no significant contribution to VVS susceptibility.

Potassium channels are involved in cardiac rhythm and vascular tone regulation. An association between the SNP rs45516097 of *KCNJ5* (a gene encoding one of the potassium channel proteins) and VVS was identified when compared 74 tilt-positive patients without cardiac, endocrine, or neurological disorders and 208 healthy blood donors (*p* = 0.001) [45]. No association with VVS was observed for the SNPs rs6590357, rs7118824, rs7118833, rs7102584, and rs4937391 of the *KCNJ3* gene. Negative results were also obtained in this study for the SNP rs138806839 of the *CHRM2* gene encoding acetylcholine receptors M2 (muscarinic receptors) capable of modulating muscarinic potassium channels (the data are provided in the “Other genes” section of the Table 1). Furthermore, no association of *KCNH2* rs1805123 and *KCNE1* rs1805127 with VVS was found [28].

Endothelial nitric oxide synthase (eNOS) plays a crucial role in the regulation of vascular tone, blood flow, and BP. However, there were no association between the polymorphic regions of the *eNOS* gene and VVS [28].

Association of VVS with polymorphic variants of other candidate genes whose products are involved in regulation of vascular tone and BP (the SNPs rs4646994 of the *ACE* gene encoding angiotensin-converting enzyme [33,41], rs699 of the angiotensin gene *AGT*, rs5186 of the *AGTR1* gene encoding angiotensin II [41], rs10478694 of the endothelin 1 gene *EDN1* [48], and rs5333 of the endothelin receptor type A gene *EDNRA* [48]) have also been studied, but no association with VVS has been revealed. However, the 4A polymorphism of the *EDN1* gene (rs10478694) was shown to be associated with a positive head-up tilt test response (*p* = 0.048). Carriers of this allele have increased endothelin-1 production. Since endothelin-1 has a vasoconstrictive effect and is expected to prevent VVS, researchers suggest that its involvement in the development of VVS is not confined to affecting vascular tone and is possibly related to a different mechanism [48].

Therefore, studies focusing on the role of individual candidate genes in VVS patients are rather controversial, which can be explained by small sample size and ethnic differences in the groups being compared, as well as the fact that it is rather labor-intensive to diagnose this condition. Furthermore, patients with VVS subdivided into tilt-positive or tilt-negative were used as controls in a number of studies, or patients with typical and atypical syncope were compared. A plausible reason for non-reproducibility of the results is that the control groups were formed from conditionally healthy individuals without any prior diagnostics, which is especially important because of the high frequency of VVS (up to 25%) in the general population. Meanwhile, the association data for a number of genes (such as serotoninergic system genes, potassium channel genes, most vascular-tone-regulating genes, etc.) have been obtained in a single study and need to be reproduced. Taking into account the complex pathogenesis and heterogeneous clinical course of VVS, it is fair to assume that this disorder is polygenic. However, none of the studies analyzed the overall contribution of candidate genes to its development.

## 4. Genome-Wide Studies

Unlike the “candidate gene” approach when a hypothesis regarding potential involvement of a gene in phenotype is put forward according to its nature and function of the gene product (the “phenotype-to-gene” approach), the genome-wide searching for genes involved in disease/phenotype development employs panels of genetic markers with known chromosomal localization. These panels can be used to identify the genomic regions where these genes localize, and then search for the genes directly associated with the phenotype within these genomic loci is performed. This approach can be defined as the “genome-to-gene” approach. Polymorphic variants distributed over the entire genome more or less uniformly serve as genetic markers. Most typically, those are SNPs. Insertions/deletions (indels) and mini- or microsatellite repeats are also analyzed rather commonly. Microarrays and other modern technologies allow one to simultaneously identify as many as several dozen thousand to several million polymorphisms within a single sample.

Genome-wide studies were originally applied to analyze the linkage between a disease and chromosomal loci in families where several members had this disease. Later, genome-wide association studies (GWAS) were used as a more powerful tool for studying human genetic architecture.

The first genome-wide study of VVS was conducted by linkage analysis in a large family presumably with autosomal dominant inheritance (30 affected individuals with VVS over three generations) using microarray-based SNP genotyping data [25]. In this family, significant linkage to the locus on chromosome 15q26 was revealed for VVS; the logarithm of odds score was 3.28. Sequencing of the *SLCO3A1*, *ST8SIA2* and *NR2F2* candidate genes residing within the linkage interval detected no mutations. Linkage to the chromosome 15q26 region was excluded in two additional large families, suggesting that different genes may be relevant in the development of VVS in these families. 

Demir et al. [54] conducted the first GWAS of VVS based on comparing the copy number variations (CNV) of repetitive genomic regions sized from one thousand to several million base pairs. Due to the greater genome coverage ensured by CNV compared to SNP markers, CNV is an important source of genetic variability and is regarded as an alternative type of DNA marker. The small study involved 16 subjects from four families with familial VVS; 13 of these subjects had a history of recurrent syncope and positive head-up tilt test response, while 3 subjects had no history of syncope. Twenty-six CNV variants whose presence in the genome differed significantly in patients with VVS compared to healthy subjects (*p* < 0.05) was revealed. In patients with VVS, the CNV segments were longer than those in healthy controls. However, the copy number presumably is not directly related to the pathophysiology of syncope, since no identical CNVs specific for individuals diagnosed with VVS have been identified.

The accuracy of assessments made in association studies directly depends on the number of DNA samples being studied. It is noteworthy that both patients and controls need to belong to the same ethnic group. A large-scale GWAS for syncope and collapse was conducted in 2020 [55]. This study used UK Biobank metadata [56], containing detailed information on the health status and genotyping results of more than 500,000 subjects. A total of 805,426 SNPs were examined as potential genetic markers. A British population was enrolled in the study: a group consisting of 9163 patients with syncope or collapse according to the International Classification of Disease (ICD-9, code 780.2, and ICD-10, code R55) and 399,798 healthy controls.

A new locus on chromosome 2q32.1 associated with VVS was identified at a significance level complying with modern requirements of GWAS results (the Bonferroni-adjusted *p* value for 1,000,000 comparisons needs to be <5 × 10^−8^). The *p* value for the lead SNP rs12465214 was 5.8 × 10^−15^; for the other four SNPs in this locus (rs7593266, rs17582219, rs12621296, and rs2219224), the *p* value ranged from 1.0 × 10^−9^ to 3.3 × 10^−8^. Carriership of the rs12465214*C risk allele was characterized by a hazard ratio of 1.13 (95% confidence interval 1.10–1.17) [55].

Results of this study were validated using the Danish Neonatal Screenings Biobank database [57]. Samples from 2352 subjects suffering from syncope and 51,929 controls were selected for GWAS analysis, which confirmed the association of the rs12465214*C allele with syncope (*p* = 8.82 × 10^−6^).

Functional annotation identified 26 genes in the locus associated with a risk of syncope [55]; the researchers believe that the *ZNF804A* gene encoding zinc finger protein 804A is the most plausible candidate gene associated with the risk of developing VVS. The SNP rs12465214 resides at a distance of approximately 250 kb from this gene and affects its expression as shown by the transcriptome analysis also conducted in [55]. Using quantitative polymerase chain reaction (q-PCR), the authors demonstrated that the *ZNF804A* gene was preferentially expressed in the brain, cerebral arteries, and endocrine tissue rather than in the cardiac muscle tissue [55].

The *ZNF804A* gene is known to be associated with schizophrenia and bipolar disorder [58,59]. Its protein product regulates processing of mRNA precursors and expression of genes, associated with synaptic transmission and development of the nervous system [60]. Based on these data, the search for associations of the VVS-associated SNPs rs12465214, rs7593266, rs17582219, rs12621296 and rs2219224 with schizophrenia was performed [55] using the GWAS data for schizophrenia [61]. However, none of these SNPs were associated with schizophrenia at a nominal significance level [55].

Enhanced expression in patients with VVS was shown for other genes encoding zinc finger proteins (ZNF28, ZNF845 and ZNF146) in a study involving a Chinese cohort of children with VVS- and age-matched controls [62].

Therefore, convincing findings on the association of the rs12465214*C risk allele residing on chromosome 2 in close proximity to the *ZNF804A* gene encoding zinc finger protein 804A have been obtained and validated using GWASs on two large ethnically homogeneous cohorts [55]. This protein contains an N-terminal C2H2-type zinc finger domain (Cys2-His2) [63]. Such domains are typical of classical transcription factors [64].

However, this study has serious limitations related to the fact that the definition of the disease was given using the ICD-10 code “syncope and collapse,” without differentiating between syncope subtypes. Since VVS is the most common type of syncope [65], there is hope that the identified associations describe the genetic nature of this specific subtype. However, this assumption needs to be verified experimentally.

## 5. Conclusions

The question regarding the genetic nature of VVS was raised in the late 1980s, and is still far from being fully answered.

Data on the inheritance of VVS accumulated thus far are indicative of its familial aggregation; the genetic component can vary from a monogenic Mendelian inheritance pattern to polygenic inherited predisposition. The observed genetic heterogeneity can be correlated with both the diversity of VVS triggers and its clinical heterogeneity.

Both genetic and external factors contribute to the development of VVS; they can play a role in monogenic (resulting in incomplete penetrance) and polygenic syncope. The effect of external and epigenetic factors makes polygenic VVS a conventional complex (multifactorial) disorder. Interplay between these factors is responsible for the complex inheritance pattern. Since the effect of external factors can modulate epigenetic processes, these mechanisms cannot be fully delineated.

While the familial analysis data are rather convincing, the conventional approach to searching for the genes involved in syncope development by analyzing the association with individual candidate genes (selected according to VVS pathophysiology and function of the gene product) has not yielded unambiguous results yet. Although the range of candidate genes is rather broad, genes whose association with VVS that can be confirmed using independent samples or at least obtained by comparing large cohorts of patients and controls sufficient to perform robust statistical analysis are yet to be discovered.

The “genome-to-gene” approach has been used to analyze genetic architecture in a few studies and has yielded fundamentally important results. The genome-wide linkage analysis of VVS in several multiplex families with autosomal dominant inheritance patterns revealed the mutant locus 15q26 in only one family [25]. These data argue convincingly in favor of the genetic heterogeneity of VVS. In 2020, a GWAS involving large heterogeneous study groups (patients with syncope and collapse vs. healthy controls) revealed a single polymorphic variant (rs12465214) associated with the disease at a genome-wide significance level in the locus 2q32.1, which was later validated using an independent sample [55]. At first glance, this finding is not consistent with the genetic heterogeneity of VVS, to say nothing of the group of syncope and collapse patients that is more heterogeneous in terms of clinical characteristics. Further research is needed to resolve this controversy. Today, it is only fair to state with a high degree of probability that the phenomenon of observing a single syncope-associated locus (while its genetic heterogeneity has been established) is attributed to the key functions of the gene closely linked to the rs12465214.

Hadji-Turdeghal et al. [55] believe that the association of the SNP rs12465214 with syncope is most likely due to the fact that it resides in close proximity to the *ZNF804A* gene in locus 2q32.1. The C2H2 transcription factor containing zinc finger protein 804A (ZNF804A) encoded by the *ZNF804A* gene can play a pivotal role in the protein–protein interaction network by participating in various regulatory and signaling pathways involved in syncope development.

We believe, however, that it is not the only possible interpretation of the results. When discussing localization of the lead SNP associated with syncope, rs12465214, on chromosome 2, Hadji-Turdeghal et al. [55] classified it as an intergenic variant. However, the SNP rs12465214 actually resides in the shared intron of two non-coding RNA genes, *LOC105373776* and *LOC102724340*, in the positive and negative DNA strands, respectively. Although the functions of these genes are unknown, we presume that these very genes might be responsible for the role of the 2q32.1 region as a locus associated with the risk of syncope development. As reported in the NCBI database [66], these genes produce several transcript variants which are long non-coding RNAs (lncRNAs). Residing in the intron, the SNP rs12465214 can affect splicing of the transcripts of these genes, thus altering the composition of lncRNAs. According to modern views, lncRNAs play a crucial role in regulating of the expression of numerous genes in various biological and pathophysiological contexts (in particular, in neuronal dysfunction and immune response) [67]. It is quite reasonable to assume that lncRNAs are involved in the formation of the genetic architecture of syncope.

Identifying genetic and epigenetic factors involved in VVS has proven to be a promising field of research, not only to improve knowledge of risk factors for VVS, which could be of help for prevention, but also to improve the understanding of the pathophysiology of syncope subtypes, and to optimize and personalize the treatment of patients with syncope in the future.

## Data Availability

Not applicable.
